# Supplementary material for: Calculating age-specific prevalence rates of female genital mutilation / cutting (FGM/C) for use as an input variable in extrapolation calculations and as predictors of future prevalence in countries of origin
Source: PLoS One. 2025 Feb 6;20(2):e0317845. doi: 10.1371/journal.pone.0317845 (PMC11801577; doi:10.1371/journal.pone.0317845)
Supplement: S1 Data — (DOCX) [file pone.0317845.s001.docx]

Supplemental Data Table 1: FGM/C prevalence extracted from 120 nationally representative surveys sorted by country and year of survey

| **Country** | **Survey** | **Age 15–19 (%)** | **Age 20–24 (%)** | **Age 25–30 (%)** | **Age 30 –34 (%)** | **Age 35–39 (%)** | **Age 40–44 (%)** | **Age 45–49 (%)** |
| --- | --- | --- | --- | --- | --- | --- | --- | --- |
| Benin | DHS 2001 | 12.1 | 13.4 | 16.9 | 18.4 | 18.3 | 25.1 | 23.7 |
| Benin | DHS 2006 | 7.9 | 9.9 | 13.6 | 14.3 | 16.3 | 17 | 15.8 |
| Benin | DHS 2011-12 | 2.0 | 4.2 | 6.9 | 9.8 | 10.4 | 11.5 | 12 |
| Benin | MICS 2014 | 2.4 | 6.3 | 9.7 | 11.5 | 13.1 | 12.4 | 18.1 |
| Burkina Faso | DHS 1998-99 | 64.2 | 70.7 | 75.0 | 73.7 | 74.1 | 76.7 | 74.1 |
| Burkina Faso | DHS 2003 | 65.0 | 76.2 | 79.2 | 79.4 | 81.6 | 83.1 | 83.6 |
| Burkina Faso | MICS 2006 | 59.7 | 69.9 | 72.6 | 77.2 | 79.8 | 81.1 | 80.5 |
| Burkina Faso | DHS 2010 | 57.7 | 69.8 | 77.5 | 82.8 | 85.2 | 88.2 | 89.3 |
| Burkina Faso | DHS 2021 | 32.2 | 46.1 | 54.5 | 64.1 | 72.2 | 77.2 | 82.5 |
| CAR | DHS 1994-95 | 34.6 | 42.7 | 44.3 | 44.1 | 47.5 | 51.4 | 53.1 |
| CAR | MICS 2000 | 27.2 | 33.8 | 35.6 | 39.9 | 43.3 | 41.5 | 41.9 |
| CAR | MICS 2006 | 18.7 | 24.0 | 26.0 | 27.3 | 29.8 | 34.6 | 31.8 |
| CAR | MICS 2010 | 17.9 | 22.1 | 24.5 | 25.5 | 28.4 | 29.9 | 33.8 |
| CAR | MICS 2018-19 | 17.3 | 19.0 | 22.0 | 21.9 | 25.3 | 29.1 | 24.8 |
| Chad | DHS 2004 | 43.4 | 45.8 | 45.2 | 43.5 | 46.2 | 46.1 | 45.9 |
| Chad | MICS 2010 | 41.0 | 43.0 | 46.1 | 45.2 | 45.7 | 44.3 | 47.6 |
| Chad | MICS 2014-15 | 31.8 | 38.1 | 41.5 | 40.7 | 40.4 | 42.2 | 39.4 |
| Chad | MICS 2019 | 28.9 | 33.2 | 37.1 | 36.7 | 34.7 | 35.5 | 39.2 |
| Côte d'Ivoire | DHS 1998-99 | 41.2 | 42.7 | 42.4 | 49.0 | 44.5 | 51.4 | 51.0 |
| Côte d'Ivoire | AIS 2005 | 39.4 | 38.7 | 41.9 | 42.5 | 44.7 | 44.1 | 48.9 |
| Côte d'Ivoire | MICS 2006 | 28.0 | 33.5 | 37.6 | 42.8 | 43.8 | 40.8 | 39.7 |
| Côte d'Ivoire | DHS 2011-12 | 31.3 | 35.1 | 36.8 | 40.3 | 45.4 | 44.6 | 46.9 |
| Côte d'Ivoire | MICS 2016 | 27.4 | 33.6 | 40.0 | 40.8 | 40.0 | 42.9 | 41.5 |
| Egypt | DHS 1995 | 98.1 | 98.3 | 97.0 | 95.8 | 96.7 | 97.2 | 96.8 |
| Egypt | DHS 2000 | 99.1 | 97.4 | 97.2 | 96.7 | 97.4 | 96.9 | 97.9 |
| Egypt | DHS 2003 | 96.8 | 97.4 | 97.3 | 96.5 | 96.4 | 96.5 | 98.0 |
| Egypt | DHS 2005 | 96.4 | 95.8 | 95.1 | 95.9 | 95.9 | 96.0 | 96.3 |
| Egypt | DHS 2008 | 93.8 | 93.6 | 94.3 | 95.7 | 97.1 | 96.8 | 96.7 |
| Egypt | DHS 2014 | 87.6 | 87.5 | 90 | 93.3 | 94.8 | 95.1 | 95.0 |
| Egypt | EHIS 2015 | 69.6 | 81.6 | 89.2 | 92.6 | 95.4 | 94.9 | 97.1 |
| Eritrea | DHS 1995 | 90.4 | 94.4 | 94.9 | 95.6 | 97 | 95.9 | 97.1 |
| Eritrea | DHS 2002 | 78.3 | 87.9 | 90.8 | 93.4 | 92.6 | 94.1 | 95.0 |
| Eritrea | EPHS 2010 | 68.8 | 79.1 | 84.9 | 88.4 | 90.4 | 93.0 | 93.1 |
| Ethiopia | DHS 2000 | 70.7 | 78.3 | 81.4 | 86.1 | 83.6 | 85.8 | 86.8 |
| Ethiopia | DHS 2005 | 62.1 | 73.0 | 77.6 | 78.0 | 81.2 | 81.6 | 80.8 |
| Ethiopia | DHS 2016 | 47.1 | 58.6 | 67.6 | 76.9 | 74.6 | 72.6 | 78.7 |
| Gambia | MICS 2005-06 | 79.9 | 78.2 | 77.2 | 78.4 | 79.5 | 77.7 | 74.2 |
| Gambia | MICS 2010 | 77.1 | 76.8 | 77.5 | 74.6 | 73.1 | 75.3 | 79.0 |
| Gambia | DHS 2013 | 76.3 | 74.1 | 73.5 | 73.9 | 76.8 | 74.9 | 75.9 |
| Gambia | MICS 2018 | 75.0 | 77.0 | 76.0 | 76.4 | 74.7 | 75.5 | 73.9 |
| Gambia | DHS 2019-20 | 72.6 | 71.7 | 74.8 | 71.3 | 72.1 | 71.6 | 74.0 |
| Ghana | DHS 2003 | 3.3 | 3.8 | 6.4 | 6.3 | 6.7 | 5.5 | 7.9 |
| Ghana | MICS 2006 | 1.4 | 2.3 | 2.7 | 5.7 | 5.7 | 5.1 | 7.4 |
| Ghana | MICS 2011 | 1.5 | 1.8 | 3.0 | 4.4 | 5.5 | 6.6 | 6.4 |
| Ghana | MICS 2017-18 | 0.6 | 1.5 | 1.8 | 3.2 | 3.0 | 3.6 | 4.9 |
| Guinea | DHS 1999 | 96.6 | 98.5 | 99.1 | 99.1 | 99.1 | 99.3 | 99.5 |
| Guinea | DHS 2005 | 89.3 | 94.6 | 96.6 | 97.4 | 98.6 | 98.1 | 99.5 |
| Guinea | DHS 2012 | 94.0 | 94.9 | 97.9 | 98.3 | 98.8 | 98.8 | 99.6 |
| Guinea | MICS 2016 | 94.5 | 96.5 | 97.3 | 97.3 | 96.6 | 99.1 | 98.7 |
| Guinea | DHS 2018 | 91.7 | 94.4 | 95.0 | 94.7 | 95.7 | 96.6 | 97.7 |
| Guinea Bissau | MICS 2006 | 43.5 | 43.0 | 43.5 | 46.5 | 48.6 | 47.4 | 40.7 |
| Guinea Bissau | MICS 2010 | 48.4 | 49.2 | 51.0 | 49.8 | 49.0 | 54.1 | 50.3 |
| Guinea Bissau | MICS 2014 | 41.9 | 45.9 | 47.7 | 46.7 | 40.5 | 47.5 | 45.2 |
| Guinea Bissau | MICS 2018-19 | 48.3 | 54.1 | 53.5 | 52.4 | 55.3 | 50.0 | 50.4 |
| Iraq | MICS 2011 | 4.9 | 7.5 | 9.1 | 9.1 | 9.7 | 9.4 | 10.3 |
| Iraq | MICS 2018 | 3.5 | 6.7 | 5.7 | 9.3 | 9.7 | 12.0 | 9.3 |
| Kenya | DHS 1998 | 26 | 32.2 | 40.4 | 40.9 | 49.3 | 47.4 | 47.5 |
| Kenya | DHS 2003 | 20.3 | 24.8 | 33.0 | 38.1 | 39.7 | 47.5 | 47.7 |
| Kenya | DHS 2008-09 | 14.6 | 21.1 | 25.3 | 30.0 | 35.1 | 39.8 | 48.8 |
| Kenya | DHS 2014 | 11.4 | 14.7 | 18.0 | 22.9 | 27.8 | 32.1 | 40.9 |
| Kenya | DHS 2022 | 9.1 | 9.9 | 13.2 | 16.1 | 18.7 | 23.8 | 23.1 |
| Liberia | DHS 2013 | 31.1 | 39.8 | 50.1 | 60.3 | 60.3 | 63.3 | 72.4 |
| Liberia | DHS 2019-20 | 20.1 | 21.3 | 26.4 | 36.6 | 43.8 | 46.6 | 54.0 |
| Mali | DHS 1995-96 | 92.5 | 94.2 | 93.9 | 94.8 | 93.9 | 94.2 | 92.4 |
| Mali | DHS 2001 | 91.2 | 91.3 | 91.9 | 92.1 | 92.3 | 91.2 | 91.0 |
| Mali | DHS 2006 | 84.7 | 84.5 | 86.7 | 84.2 | 84.9 | 86.2 | 85.8 |
| Mali | MICS 2009-10 | 87.7 | 88.2 | 87.9 | 89.1 | 90.2 | 89.4 | 88.5 |
| Mali | DHS 2012-13 | 90.3 | 91.5 | 92.7 | 90.8 | 90.6 | 92.7 | 92.1 |
| Mali | MICS 2015 | 83.1 | 82.7 | 82.9 | 81.0 | 82.1 | 84.1 | 83.9 |
| Mali | DHS 2018 | 86.2 | 87.4 | 90.9 | 89.6 | 88.1 | 90.8 | 89.1 |
| Mauritania | DHS 2000-01 | 65.9 | 71.1 | 73.4 | 74.2 | 71.7 | 76.5 | 68.6 |
| Mauritania | MICS 2007 | 68.0 | 68.7 | 73.5 | 73.5 | 76.7 | 77.8 | 77.8 |
| Mauritania | MICS 2011 | 65.9 | 66.2 | 67.4 | 71.3 | 72.0 | 76.2 | 75.2 |
| Mauritania | MICS 2015 | 62.5 | 64.1 | 67.9 | 65.5 | 69.1 | 71.5 | 72.5 |
| Mauritania | DHS 2019-21 | 55.8 | 60.8 | 67.8 | 66.8 | 66.5 | 68.3 | 73.8 |
| Niger | DHS 1998 | 5.0 | 4.8 | 4.3 | 5.3 | 3.8 | 3.3 | 3.3 |
| Niger | DHS 2006 | 1.9 | 1.6 | 2.4 | 2.1 | 2.9 | 2.9 | 2.8 |
| Niger | DHS 2012 | 1.4 | 2.1 | 1.9 | 1.7 | 3.0 | 2.3 | 1.4 |
| Nigeria | DHS 2003 | 12.9 | 17.0 | 20.8 | 19.4 | 22.2 | 22.2 | 28.4 |
| Nigeria | MICS 2007 | 19.6 | 22.0 | 24.6 | 26.7 | 29.7 | 31.2 | 40.3 |
| Nigeria | DHS 2008 | 21.7 | 26.4 | 28.9 | 32.8 | 33.9 | 36.4 | 38.1 |
| Nigeria | MICS 2011 | 18.7 | 21.5 | 26.1 | 29.7 | 31.5 | 34.9 | 38.0 |
| Nigeria | DHS 2013 | 15.3 | 21.7 | 22.9 | 27.4 | 30.4 | 33.0 | 35.8 |
| Nigeria | MICS 2016-17 | 12.3 | 15.4 | 16.9 | 20.1 | 21.3 | 24.4 | 27.6 |
| Nigeria | DHS 2018 | 13.7 | 15.9 | 18.0 | 19.7 | 21.9 | 26.7 | 31.0 |
| Nigeria | MICS 2021 | 6.7 | 9.8 | 12.8 | 15.7 | 20.3 | 25.2 | 29.1 |
| Senegal | DHS 2005 | 24.8 | 28.0 | 28.4 | 30.1 | 30.5 | 30.3 | 30.6 |
| Senegal | DHS 2010-11 | 24 | 24.3 | 26.1 | 24.9 | 29.0 | 26.9 | 28.5 |
| Senegal | DHS 2014 | 21.1 | 21.5 | 27.9 | 28.2 | 24.9 | 27.2 | 25.7 |
| Senegal | DHS 2015 | 22.2 | 22.3 | 24.6 | 25.1 | 24.2 | 29.4 | 27.8 |
| Senegal | DHS 2016 | 20.6 | 22.6 | 22.5 | 23.3 | 25.0 | 22.2 | 26.0 |
| Senegal | DHS 2017 | 21.3 | 23.3 | 24.5 | 23.1 | 27.2 | 26.8 | 26.1 |
| Senegal | DHS 2018 | 21.4 | 21.9 | 25.1 | 25.4 | 22.9 | 23.8 | 24.5 |
| Senegal | DHS 2019 | 23.7 | 24.2 | 27.5 | 25.4 | 25.9 | 27.9 | 23.0 |
| Sierra Leone | MICS 2005 | 81.1 | 93.6 | 96.2 | 96.4 | 97.5 | 97.1 | 97.1 |
| Sierra Leone | DHS 2008 | 75.5 | 89.4 | 95.2 | 94.9 | 96.4 | 96.1 | 95.9 |
| Sierra Leone | MICS 2010 | 70.1 | 86.9 | 92.2 | 93.2 | 95.8 | 95.3 | 96.4 |
| Sierra Leone | DHS 2013 | 74.3 | 87.5 | 93.2 | 95.8 | 97.5 | 97.4 | 97.8 |
| Sierra Leone | MICS 2017 | 64.3 | 85.7 | 90.9 | 94.5 | 96.4 | 97.5 | 96.3 |
| Sierra Leone | DHS 2019 | 61.1 | 81.0 | 88.1 | 90.7 | 93.1 | 93.5 | 94.9 |
| Somalia | MICS 2006 | 96.7 | 97.9 | 97.9 | 98.8 | 98.9 | 97.9 | 99.1 |
| Somalia | MICS 2011 | 97.3 | 98.5 | 97.9 | 98.7 | 98.3 | 97.6 | 97.8 |
| Somalia | SHDS 2020 | 98.8 | 98.6 | 99.5 | 99.7 | 99.4 | 99.6 | 99.8 |
| Sudan | DHS 1989-90 | 86.8 | 89.7 | 88.6 | 89.7 | 89.0 | 89.0 | 90.9 |
| Sudan | MICS 2010 | 83.7 | 86.8 | 89.5 | 88.3 | 89.7 | 89.8 | 89.1 |
| Sudan | MICS 2014 | 81.7 | 85.7 | 87.6 | 88.0 | 86.6 | 91.4 | 91.8 |
| Tanzania | DHS 1996 | 13.5 | 15.9 | 19.6 | 20.8 | 18.7 | 21.3 | 22.2 |
| Tanzania | DHS 2004-05 | 9.1 | 13.7 | 15.2 | 16.0 | 16.0 | 18.8 | 22.9 |
| Tanzania | DHS 2010 | 7.1 | 11.0 | 11.7 | 19.1 | 21.6 | 22.2 | 21.5 |
| Tanzania | DHS 2015-16 | 4.7 | 7.3 | 8.1 | 12.8 | 13.4 | 15.4 | 18.7 |
| Tanzania | DHS 2022 | 3.7 | 6.2 | 6 | 9.9 | 10.3 | 12.1 | 16.8 |
| Togo | MICS 2006 | 1.3 | 2.5 | 6.2 | 6.5 | 9.4 | 10.0 | 9.5 |
| Togo | MICS 2010 | 1.1 | 2.4 | 3.7 | 4.7 | 6.4 | 5.2 | 6.7 |
| Togo | DHS 2013-14 | 1.8 | 2.2 | 3.8 | 4.9 | 6.5 | 8.6 | 10.2 |
| Togo | MICS 2017 | 1.4 | 1.9 | 2.4 | 3.8 | 4.1 | 4.7 | 5.4 |
| Uganda | DHS 2006 | 0.5 | 0.8 | 0.3 | 0.8 | 0.8 | 1.0 | 0.4 |
| Uganda | DHS 2011 | 1.0 | 0.8 | 1.9 | 2.1 | 1.3 | 1.7 | 1.9 |
| Uganda | DHS 2016 | 0.1 | 0.3 | 0.2 | 0.4 | 0.5 | 0.7 | 0.4 |
| Yemen | DHS 1997 | 19.3 | 22.2 | 21.3 | 22.9 | 23.6 | 25.1 | 25.0 |
| Yemen | DHS 2013 | 16.4 | 16.8 | 16.4 | 20.5 | 23.1 | 22.1 | 22.8 |

Supplemental Data Table 2: Ethiopian Diaspora Population in the US in 2019 and FGM/C prevalence based on method proposed by three different researchers.

|  | **Age  0–4** | **Age  5–9** | **Age**  **10–14** | **Age**  **15–19** | **Age**  **20–24** | **Age**  **25–29** | **Age**  **30–34** | **Age**  **35–39** | **Age**  **40–44** | **Age**  **45–49** | **Age**  **50–54** | **Age**  **55–59** | **Age**  **60–64** | **Age**  **65–69** | **Age**  **70–74** | **Age**  **75–79** | **Age 80+** | **Total** |
| --- | --- | --- | --- | --- | --- | --- | --- | --- | --- | --- | --- | --- | --- | --- | --- | --- | --- | --- |
| **Target population (TP) extracted from ASC 2015-19** | | | | | | | | | | | | | | | | | | |
| Ethiopian Population | 13,529 | 13,912 | 11,610 | 10,886 | 9,453 | 12,170 | 18,278 | 19,211 | 14,973 | 12,259 | 8,992 | 6,637 | 4,590 | 3,548 | 1,780 | 1,072 | 1,069 | 163,969 |
| **Prevalence data (Pr) used in each method** | | | | | | | | | | | | | | | | | | |
| Jones | 65.2% | 65.2% | 65.2% | 65.2% | 65.2% | 65.2% | 65.2% | 65.2% | 65.2% | 65.2% | 65.2% | 65.2% | 65.2% | 65.2% | 65.2% | 65.2% | 65.2% |  |
| Goldberg | 47.1% | 47.1% | 47.1% | 47.1% | 65.2% | 65.2% | 65.2% | 65.2% | 65.2% | 65.2% | 65.2% | 65.2% | 65.2% | 65.2% | 65.2% | 65.2% | 65.2% |  |
| Callaghan | 23.3% | 27.1% | 31.5% | 36.8% | 43.0% | 50.5% | 58.6% | 66.9% | 74.0% | 76.8% | 78.7% | 81.9% | 81.4% | 81.8% | 82.1% | 82.1% | 82.1% |  |
| **Impacted population resulting from each method** | | | | | | | | | | | | | | | | | | |
| Jones | 8,821 | 9,071 | 7,570 | 7,098 | 6,163 | 7,935 | 11,917 | 12,526 | 9,762 | 7,993 | 5,863 | 4,327 | 2,993 | 2,313 | 1,161 | 699 | 697 | 106,908 |
| Goldberg | 6,372 | 6,553 | 5,468 | 5,127 | 6,163 | 7,935 | 11,917 | 12,526 | 9,762 | 7,993 | 5,863 | 4,327 | 2,993 | 2,313 | 1,161 | 699 | 697 | 97,869 |
| Callaghan | 3,155 | 3,769 | 3,662 | 4,006 | 4,069 | 6,141 | 10,709 | 12,860 | 11,086 | 9,415 | 7,074 | 5,433 | 3,736 | 2,903 | 1,461 | 880 | 878 | 91,236 |
| **Impacted population segmented into two age cohorts** | | | | | | | | | | | | | | | | | | |
|  |  |  | **Age**  **0 – 14** | **Age 15+** |  |  |  |  |  |  |  |  |  |  |  |  |  |  |
| Jones |  |  | 25,461 | 81,447 |  |  |  |  |  |  |  |  |  |  |  |  |  |  |
| Goldberg |  |  | 18,393 | 79,476 |  |  |  |  |  |  |  |  |  |  |  |  |  |  |
| Callaghan |  |  | 10,586 | 80,651 |  |  |  |  |  |  |  |  |  |  |  |  |  |  |
