# Supplementary material for: Calculating age-specific prevalence rates of female genital mutilation / cutting (FGM/C) for use as an input variable in extrapolation calculations and as predictors of future prevalence in countries of origin
Source: PLoS One. 2025 Feb 6;20(2):e0317845. doi: 10.1371/journal.pone.0317845 (PMC11801577; doi:10.1371/journal.pone.0317845)
Supplement: Attachment — Submitted filename: PONE-D-24-20214_Comments.pdf [file pone.0317845.s002.pdf]

This is a well-written article, and I enjoyed reading it. The Methods behind the results have been described with due diligence, and I find them comprehensible even for readers without an epidemiology background. The following comments focus on the structure of the article.

Major comments

1. There is not a clear distinction between the Sections: the Introduction section starts with a description of the problem, and there is a logical flow; however, this section ends abruptly with a formula, which I would expect to see in the Methods section with the necessary details to understand the formula. The Methods section introduces the data sources used and has a logical flow; however, this section is very lengthy and mixes information that could be presented in the Discussion, where the results of the present study are juxtaposed with those of previous studies. Consequently, the Discussion and Limitations sections are very small.
2. Comprehension of the results will be enhanced if the following suggested amendments are performed:
   - Table 13
     - Sort the countries in increasing order of the % in Age 0 to 4. This amendment will highlight the countries with lower and higher prevalence already from younger ages.
     - Use the power of Excel to colour the cells with green/red shades for each country: the lower the prevalence below 50%, the darker the green, and the larger the prevalence above 50%, the darker the red. Ensure that green and red shades for the larger prevalences are not too dark, making it difficult to read the numbers. Note that the shading should be uniform for all countries; namely, the shading should not be based on each country's minimum and maximum prevalence, as it will make the Table misleading: minimum and maximum prevalence are considered 0% and 100%, respectively, in *all* countries, with shades getting 'whiter' for prevalences close to 50% from both 'directions'. Hence, countries with prevalences above 50% will have only red shades, and those with prevalences below 50% will have only green shades.
   - Table 12
     - Apply the same amendments suggested for Table 13; however, sorting will refer to the **Survey type** *within* each country!
     - *Optional:* First, you may sort the countries in increasing order of the average prevalence for Age 15-19, and then, sort the **Survey type** *within* each country

Minor comments

1. There are many single-sentence paragraphs in the Methods section that should be avoided.
