# Supplementary material for: Calculating age-specific prevalence rates of female genital mutilation / cutting (FGM/C) for use as an input variable in extrapolation calculations and as predictors of future prevalence in countries of origin
Source: PLoS One. 2025 Feb 6;20(2):e0317845. doi: 10.1371/journal.pone.0317845 (PMC11801577; doi:10.1371/journal.pone.0317845)
Supplement: Attachment — Submitted filename: Response to reviewers.pdf [file pone.0317845.s003.pdf]

Sean Callaghan

May 2024 submission

October 2024 resubmission

PLOS One

October 18, 2024

Dear academic editor and reviewers,

RE: Response to reviewers with regards to PONE-D-24-20214

The resubmission enclosed with this letter, ***Calculating Age-Specific Prevalence Rates of female genital mutilation / cutting (FGM/C) for use as an input variable in extrapolation calculations and as predictors of future prevalence in countries of origin***, responds to the points raised by the reviewers in late September 2024.

Firstly, I would like to thank the reviewers for their positive feedback regarding the article. Your comments have strengthened my resubmission and widened the application of the method described in the article. I have been able to make most of the edits suggested and below respond to your specific comments:

General

- The title, abstract and conclusion of the article have been updated to reflect the widened scope of the article.
- The article has been restructured into the traditional five sections – Introduction, Method, Results, Discussion and Conclusion.

Introduction

- The global migrant data set has been updated.
- Clarity was brought my explanation of the extrapolation equation.
- Clarity and detail were added to the source data section (now labelled 1.1).
- The review of previous studies (now labelled 1.2) was reworked to better articulate the limitations of both US and European studies.

Method

- The 2019 population data used in the application of the method was moved to the discussion secion.
- Heading and descriptions in the method steps were strengthened.

- The tables headers were corrected.
- Single sentences were integrated into paragraphs.

Results

- The application to the method to the 2019 population data was moved to the discussion section.
- The long table of input prevalence data was moved to supplemental data.
- The results table showing 2024 prevalence was reordered by 0-4-year-old prevalence but not colour coded as it was felt that this would not add significantly to the understanding of the results.

Discussion

- The Discussion section was reworked and expanded.
- The application of the method to the 2019 target population using Jones, Goldberg and this method was reworked and consolidated into the new section 4.1.
- More detailed calculations that underpin the comparison between the three methods was added as supplemental data.
- Application of the method in the wider context was explored in section 4.3.
- The limitations (4.4) were expanded.

With regards to the question from Reviewer #2 regarding the application of this method to Callaghan (2023): a version of this refined method is being used by the author in analysis for his PhD (ongoing), early results of which were published by the non-profit AHA Foundation in November 2023. This grey literature publication while based on the method described here did not articulate the methodology outlined in this paper.

In the attached tracked resubmission, I have noted with comments those sections that were moved around the document and then tracked the actual edits to sections of text.

We thank you for your time in considering this resubmission.

Sincerely,

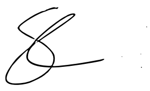

Sean Callaghan
