# Supplementary material for: Calculating age-specific prevalence rates of female genital mutilation / cutting (FGM/C) for use as an input variable in extrapolation calculations and as predictors of future prevalence in countries of origin
Source: PLoS One. 2025 Feb 6;20(2):e0317845. doi: 10.1371/journal.pone.0317845 (PMC11801577; doi:10.1371/journal.pone.0317845)
Supplement: Attachment — Submitted filename: Response to reviewers (v3).pdf [file pone.0317845.s004.pdf]

Sean Callaghan

May 2024 submission

December 2024 resubmission

PLOS One

December 27, 2024

Dear academic editor and reviewers,

RE: Response to reviewers with regards to PONE-D-24-20214R1

The resubmission enclosed with this letter, ***Calculating Age-Specific Prevalence Rates of female genital mutilation / cutting (FGM/C) for use as an input variable in extrapolation calculations*** **and as predictors of future prevalence in countries of origin**, separates the supplemental data from the manuscript as requested.

We thank you for your time in considering this resubmission.

Sincerely,

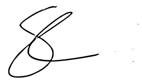

Sean Callaghan
